# Improvement of therapeutic index for brain tumors with daily image guidance

**DOI:** 10.1186/1748-717X-8-283

**Published:** 2013-12-02

**Authors:** Lisa BE Shields, James M Coons, Catherine Dedich, Maria Ragains, Kristi Scalf, Todd W Vitaz, Aaron C Spalding

**Affiliations:** 1 Norton Neuroscience Institute, Louisville, KY, USA; 2 The Brain Tumor Center, Norton Healthcare, Louisville, KY, USA; 3 The Norton Cancer Institute Radiation Center and Kosair Children’s Hospital, Louisville, KY, USA; 4 The Norton Cancer Institute Radiation Center, 676 S. Floyd St., Suite 100, Louisville, KY 40202, USA

**Keywords:** Radiation, Oncology, Brain tumor, CNS malignancy

## Abstract

**Background:**

Image-guidance maximizes the therapeutic index of brain irradiation by decreasing setup uncertainty. As dose-volume data emerge defining the tolerance of critical normal structures responsible for neuroendocrine function and neurocognition, minimizing clinical target volume (CTV) to planning target volume (PTV) expansion of targets near these structures potentially lessens long-term toxicity.

**Methods:**

We reviewed the treatment records of 29 patients with brain tumors, with a total of 517 fractions analyzed. The CTV was uniformly expanded by 3 mm to create the PTV for all cases. We determined the effect of patient specific factors (prescribed medications, weight gain, tumor location) and image-guidance technique on setup uncertainty and plotted the mean +/- standard deviation for each factor. ANOVA was used to determine significance between these factors on setup uncertainty. We determined the impact of applying the initial three fraction variation as custom PTV-expansion on dose to normal structures.

**Results:**

The initial 3 mm margin encompassed 88% of all measured shifts from daily imaging for all fractions. There was no difference (p = n.s.) in average setup uncertainty between CBCT or kV imaging for all patients. Vertical, lateral, longitudinal, and 3D shifts were similar (p = n.s.) between days 1, 2, and 3 imaging and later fractions. Patients prescribed sedatives experienced increased setup uncertainty (p < 0.05), while weight gain, corticosteroid administration, and anti-seizure medication did not associate with increased setup uncertainty. Patients with targets near OAR with individualized margins led to decreased OAR dose. No reductions to targets occurred with individualized PTVs.

**Conclusions:**

Daily imaging allows application of individualized CTV expansion to reduce dose to OAR responsible for neurocognition, learning, and neuroendocrine function below doses shown to correlate with long-term morbidity. The demonstrated reduction in dose to OAR in this study has implications for quality of life and provides the motivation to pursue custom PTV expansion.

## Introduction

The underlying goal of treating CNS malignancies is to maximize tumor eradication while preserving parenchymal brain function. Tilting the therapeutic index towards eradicating tumor cells while protecting normal tissue may be improved by reducing setup uncertainty. Image guided radiation therapy (IGRT) has the potential to improve accuracy through patient localization.

The term clinical target volume (CTV) is defined in the International Commission on Radiation Units and Measurements (ICRU) Reports as “a tissue volume that contains a gross tumor volume (GTV) which is the gross palpable or visible/demonstrable extent + and location of the malignant growth, and/or subclinical microscopic malignant disease, which has to be eliminated. This volume has to be treated adequately in order to reach the aim of therapy: cure or palliation” [1]. Planning target volume (PTV) is defined as “a geometrical concept, and it is defined to select appropriate beam sizes and beam arrangements, taking into consideration the net effect of all the possible geometrical variations and inaccuracies in order to ensure that the prescribed dose is actually absorbed in the CTV” [1]. The PTV is composed of two factors: (1) the internal margin (IM) which relies on temporal changes in position, volume, and shape of the CTV and the (2) setup margin (SM) which accounts for uncertainties in patient position and beam delivery that is inherent with fractionated irradiation [2-4]. If the margin between the CTV and PTV is too large, there is a higher likelihood of excessive radiation to normal tissue [5]. Inversely, if the margin is too small, an undesirable outcome may occur due to inadequate radiation of the target tissue. The present study highlights patient features and technical interventions which may play a role in influencing the setup margin.

We tested the following hypotheses: 1) that the use of days 1, 2, and 3 cone-beam CT scan or orthogonal kV imaging predicted patient position during the treatment course, 2) non technology related patient factors, specifically a) prescribed medications b) weight gain, c) and tumor location predicted setup uncertainty, and 3) application of custom CTV to PTV expansion from the first three fractions reduces dose to organs at risk (OAR).

## Methods

Under an IRB-approved protocol and in compliance with the Helsinki Declaration, we reviewed the treatment records of 29 patients with brain tumors immobilized with an aquaplast mask and standard base plate or an S-frame and aquaplast mask for simulation. The treatment planning CT with 1 mm neutral gantry axial slices was fused with a gadolinium-enhanced MRI with 3 mm zero tilt axial, coronal, and sagittal slices for target delineation. The CTV was uniformly expanded by 3 mm to create the PTV for all cases to minimize fusion errors. This approach is similar to a phantom study shown to have an accuracy of autofusion less than 0.5 mm [6]. Anatomic verification of deep brain electrode placement based on CT/MRI fusion has been shown to have accuracy of a similar magnitude [7]. OAR were contoured (temporal lobes, brainstem, bilateral hippocampus, cochlea, hypothalamus, and pituitary gland) and were used for inverse-planned static gantry IMRT. The equipment utilized was a Varian linear accelerator with orthogonal kilovoltage imagery.

All patients were treated with daily fractionated radiation with the dose dependent on tumor histology: 21 patients with high grade glioma received 60 Gy in 30 fractions; 4 patients with low grade glioma, 2 patients with meningioma, and 2 patients with pituitary adenoma received 50.4 Gy in 28 fractions. The number of beams and angles was chosen based on tumor location and proximity of OAR.

A total of 517 treatments were delivered with online corrections made and recorded in the vertical, lateral, and longitudinal axis. The three dimensional vector of uncertainty was calculated as the square root of the sum of the squares of each axis:

$$\;3D\;\mathrm{shift}=\sqrt{{\left(\mathrm{Vert}\right)}^{2}+{\left(\mathrm{Lat}\right)}^{2}+{\left(\mathrm{Long}\right)}^{2}}$$

Patients found to have setup uncertainty less than 3 mm were planned with the measured setup uncertainty, with no change in optimization parameters. The setup uncertainty was dependent on the localization. A bony match as opposed to a soft tissue match was utilized since the base of the skull served as a surrogate for intracranial targets. We determined the effect of patient specific factors (prescribed medications, weight gain, and tumor location) and technical interventions on setup uncertainty, and plotted the mean +/- standard deviation for each factor. Student’s t-test was used to compare between groups.

## Results

### Image Guidance Modality and Setup Uncertainty

We first determined the influence of Cone Beam Computed Tomography (CBCT) versus On Board Imaging (OBI) imaging on patient set up variability. For each of the 29 patients, shift data was recorded and analyzed for both techniques. There was no difference between techniques for shifts in the vertical (2+/-4 mm CBCT vs 2+/-2 mm OBI, p = 0.45), longitudinal (1.3+/-1.4 mm CBCT vs 1.4+/- 1.4 mm OBI, p = 0.07), or three dimensional (3.5+/-4.2 mm CBCT vs 3.6+/- 1.9 mm OBI, p = 0.52) vector averages. There was a statistical difference in the lateral vector (1.4+/-1.6 mm CBCT vs 1.7+/- 1.5 mm OBI, p < 0.001) of small magnitude. We found no clinically meaningful difference between the two imaging modalities for evaluating translational setup uncertainty.

### Setup Uncertainty First Three Treatment Days vs. Subsequent Treatments via CBCT Image Guidance

**Figure 1:**
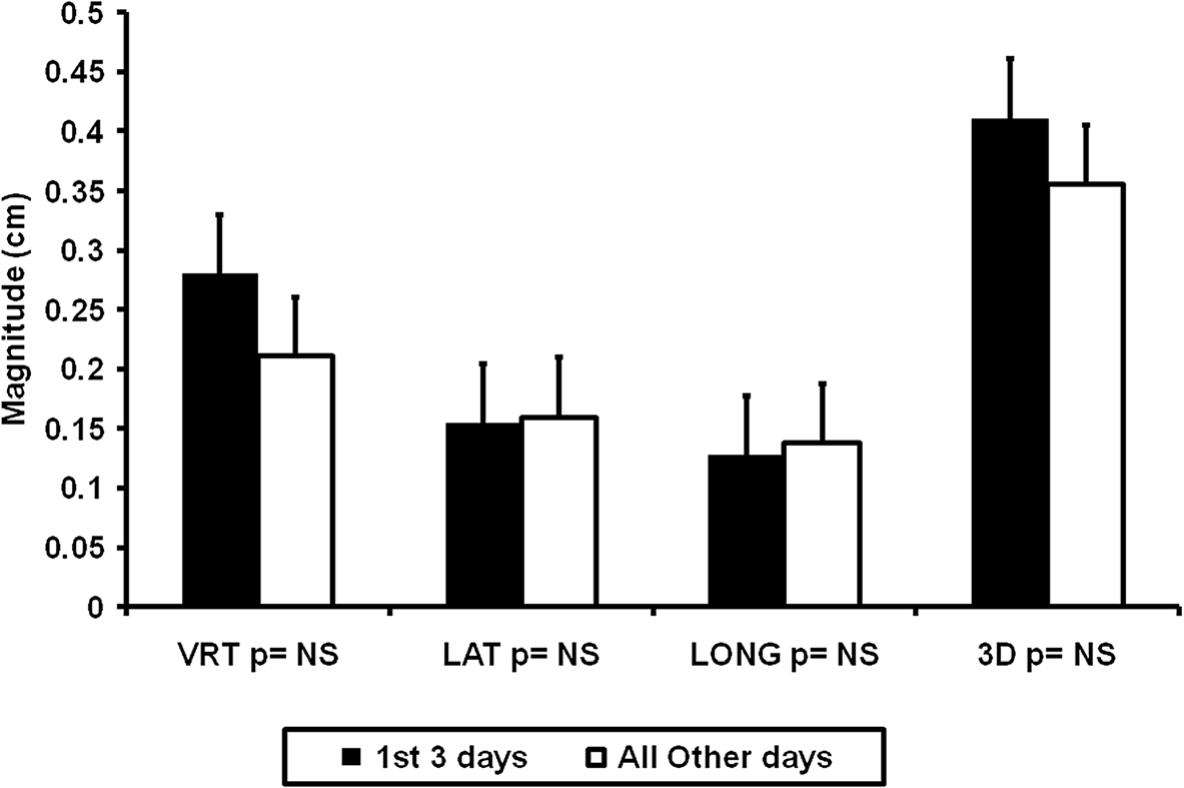
**Initial three IGRT fractions predict future patient position with CBCT.** The vertical, lateral, longitudinal, and total three dimensional setup uncertainty averages were plotted with +/- standard deviations for the first three days of treatment in black and all subsequent treatments in white. Student’s t-test was used to determine significance between groups.

We next determined whether the first three days of IGRT would predict patient setup for subsequent fractions. Patients underwent CBCT for the first three fractions and remarked before the fourth fraction. As shown in Figure 1, there was no statistical difference (p = NS) in setup uncertainty between the first three days of treatment and all subsequent treatments via CBCT image guidance. We also analyzed post treatment images to quantify intrafraction motion. For 100 fractions, the mean difference from pre-treatment to post-treatment images was 0.7 mm.

### Immobilization Device and Setup Uncertainty

**Figure 2:**
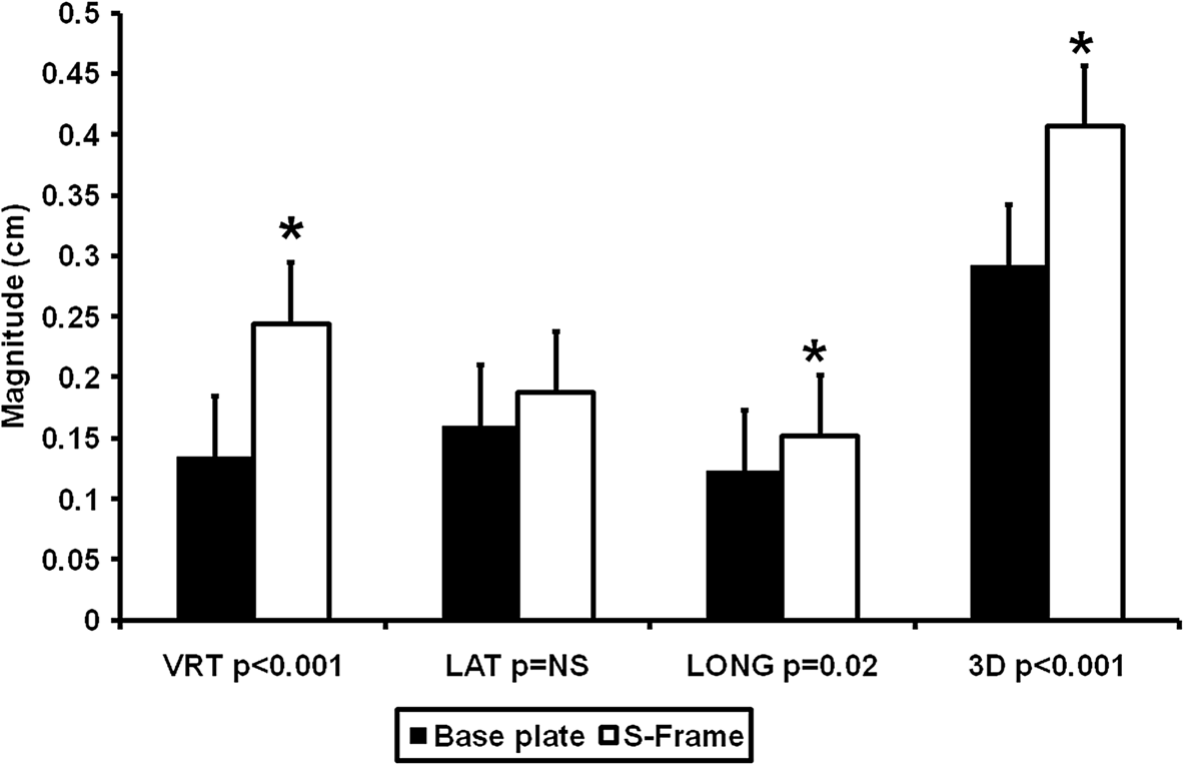
**S-frame extension contributes setup uncertainty compared with base plate immobilization.** Setup uncertainties with +/- standard deviations for patients immobilized via aquaplast mask and base plate are demonstrated in black and those immobilized via aquaplast mask and S-Frame in white. * Indicates p < 0.05 by Student’s t-test.

The use of an S-frame table extender allows the use of additional beam angles to improve isodose conformality and OAR sparing. However, there may be setup error introduced due to flexion or insertion variability. We, therefore, compared the vertical, lateral, longitudinal, and three dimensional setup uncertainty for patients immobilized via an aquaplast mask and base plate (N = 6) vs. an aquaplast mask and S-Frame (N = 23) in Figure 2. S-frame immobilization demonstrated a clinically significant increase in setup uncertainty in the vertical axis (2.5 +/- 3.7 mm S Frame vs. 1.3 +/- 0.9 mm for base plate, p < 0.001) which contributed to the observed significant difference in 3D setup variability.

### Medications and Setup Uncertainty

**Figure 3:**
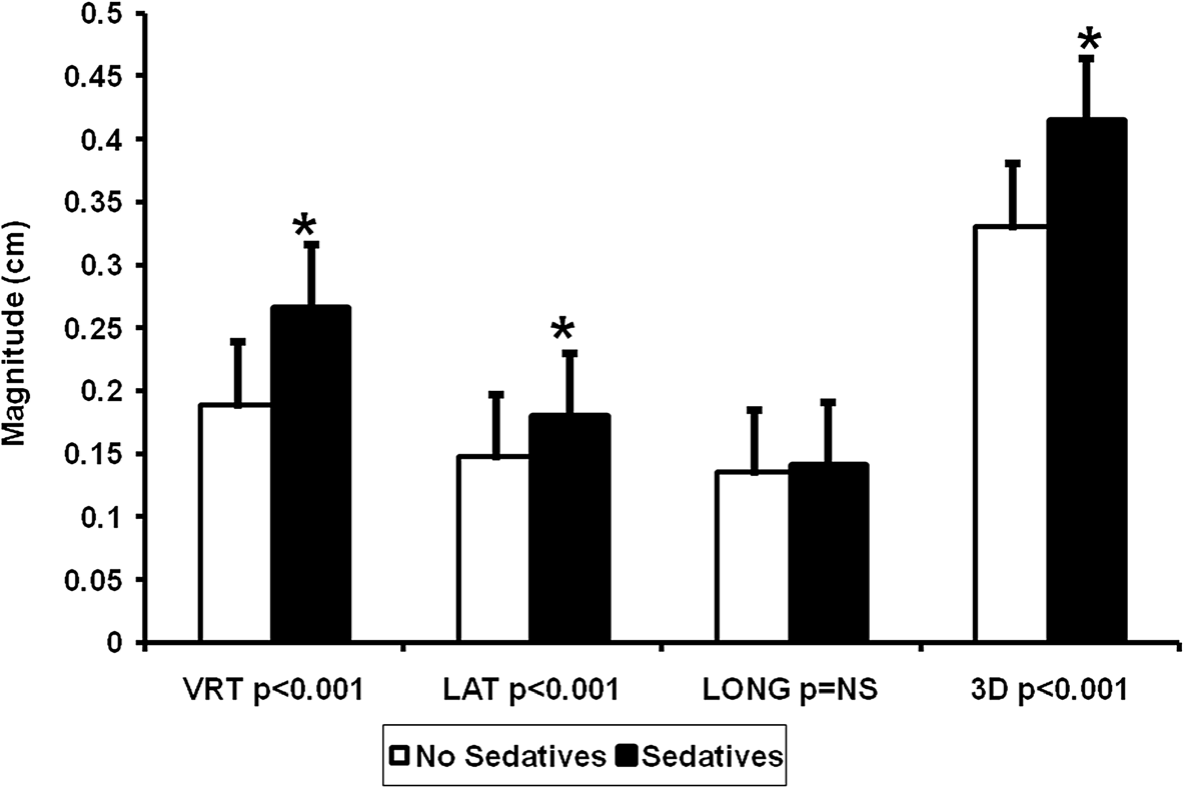
**Influence of sedatives on setup uncertainty.** Setup uncertainty data in the vertical, lateral, longitudinal, and total three dimensional setup uncertainty were plotted with +/- standard deviation. Setup uncertainty was statistically significant in patients who were prescribed sedatives, shown in white (* p < 0.05).

The time from radiation CT simulation and planning to the completion of radiation therapy can be up to eight weeks for a 30 fraction course, and patients may change during this time frame which may impact setup in a thermoplastic mask. We measured the impact of corticosteroid administration (14 yes vs. 15 no), sedative administration (11 yes vs. 18 no), anti-seizure administration (14 yes vs. 15 no), and weight change (23 stable vs. 6 with at least a 10 lb gain) on setup uncertainty. Patient medication history data were reviewed, and the status for three prescribed medications was recorded. There was no statistical difference in the proportion of patients prescribed medications and those not prescribed medications (p = NS). Neither steroid administration nor weight change was associated with differences in setup variability. However, as shown in Figure 3, patients administered sedatives had increased setup uncertainty (3D vector of 3.3+/-1.6 mm for non-sedated vs. 4.1+/-3.8 mm for sedated patients). Those receiving anti-seizure medications had statistically decreased setup uncertainty in the vertical, lateral, and longitudinal axes of minimal clinical magnitude of less than 0.5 mm in each vector.

### Tumor Location and Setup Uncertainty

**Figure 4:**
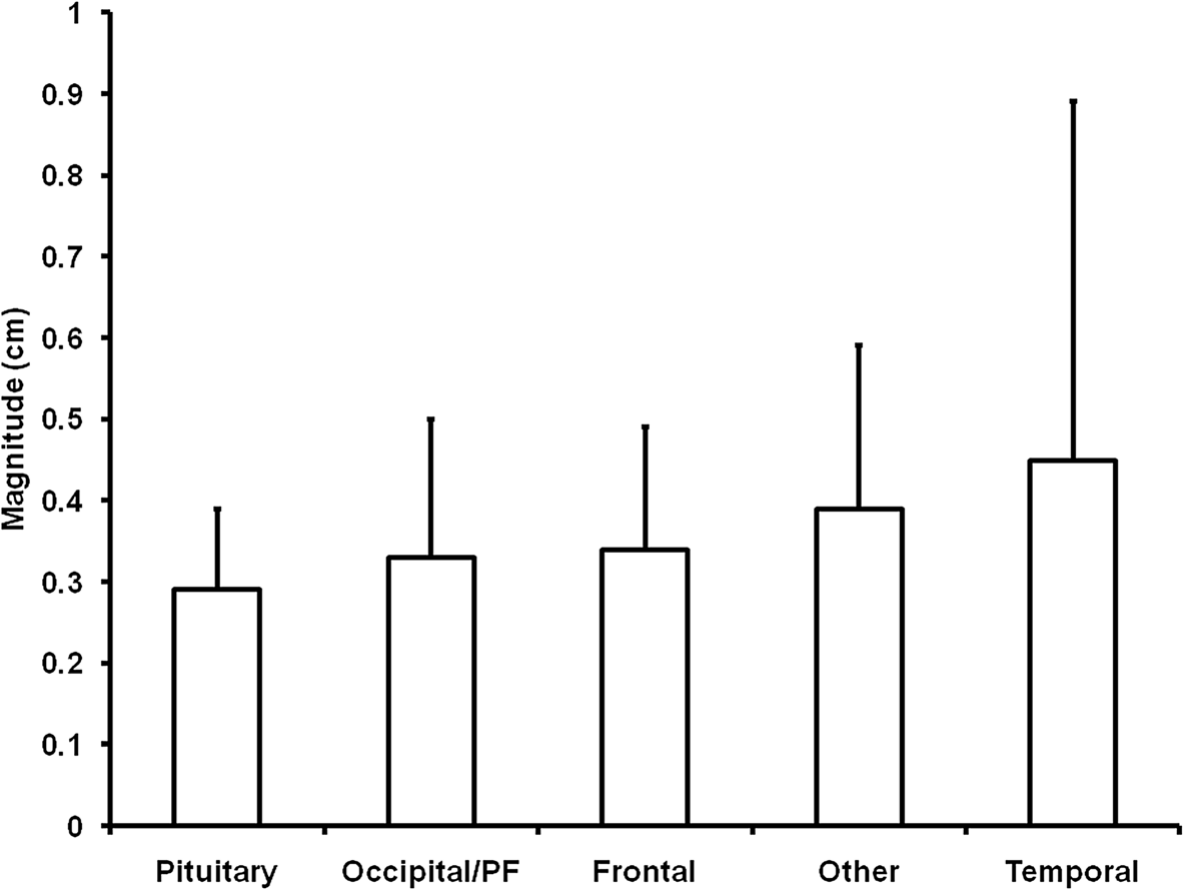
**Tumor location influences setup uncertainty.** The 3D vector +/- the standard deviation for each of the tumor locations are shown. P < 0.05 by ANOVA across all groups.

We hypothesized that central tumors would have less setup uncertainty since rotational errors would not influence location as opposed to lateral tumors. We classified targets (n = 29) according to location: Pituitary (n = 3), frontal lobe (n = 8), temporal lobe (n = 7), occipital/posterior fossa (n = 9), and other (n = 4). There was no statistical difference in the proportion of tumor location (p = NS). Figure 4 shows the 3D vector uncertainty average for the five groups with pituitary targets having the least variability (2.8+/-2.0 mm) and temporal lobe tumors demonstrating the highest (4.4+/-4.4 mm, p <0.05 across all groups by ANOVA). In this analysis, the distance from the center of the skull correlated with setup uncertainty.

### Effect of Custom PTV on Dose to OAR

**Figure 5:**
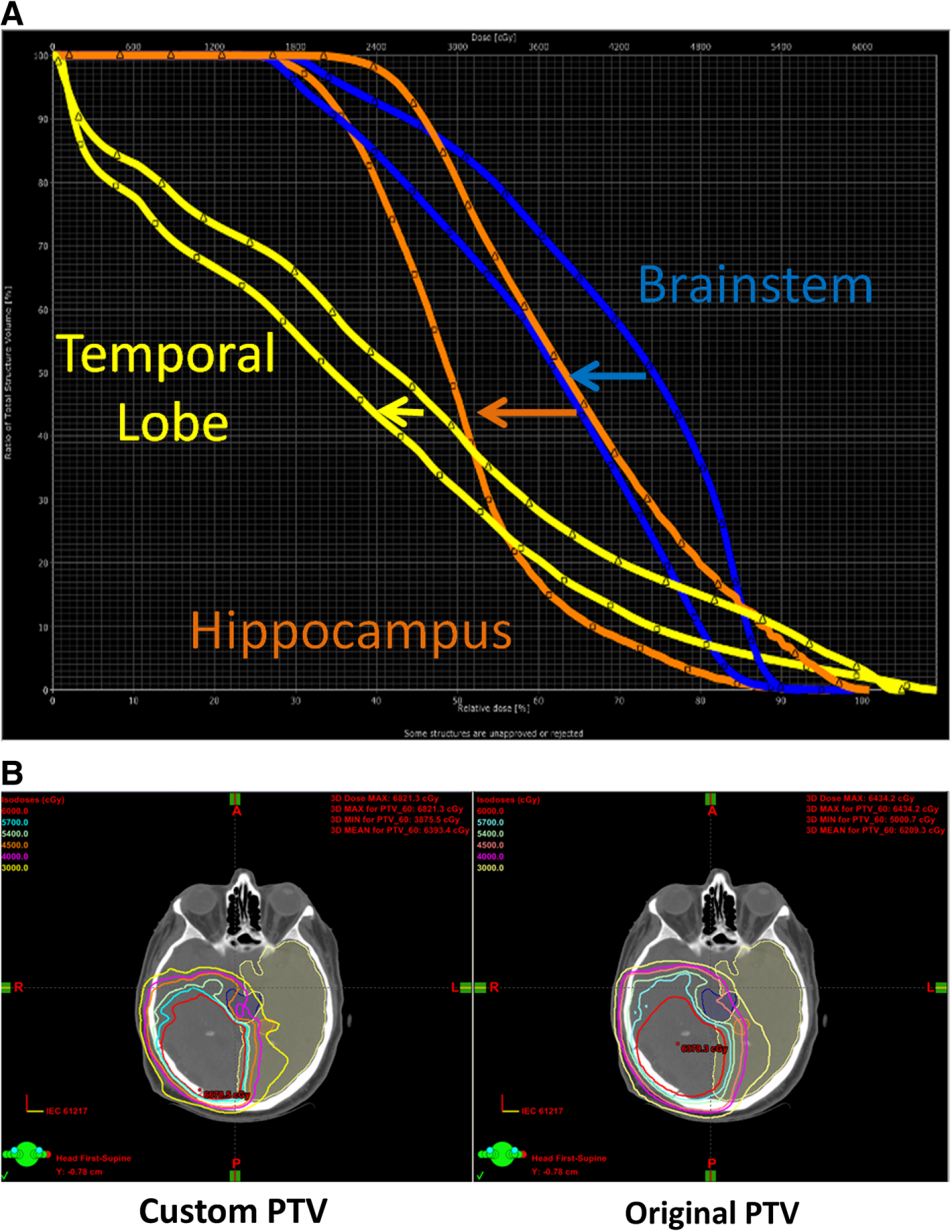
**Planning with customized PTV allows decreased dose to critical normal structures. A.** A patient with a centrally-located malignant glioma underwent replanning with a CTV to PTV expansion of 1.5 mm as measured by IGRT in the first three days of treatment. This decreased dose to the brainstem (blue, max from 5935 to 5550 cGy and mean from 4153 to 3596 cGy), hippocampus (orange, max from 6052 to 5761 cGy and mean from 3892 to 3006 cGy), and temporal lobe (yellow, max from 6550 to 6311 cGy and mean from 2623 to 2205 cGy). **B.** Axial representative isodose curves from original and reduced PTV, respectively.

Minimizing CTV to PTV expansion can lead to decreased dose to normal tissues in close proximity to radiation targets. Three of the 29 patients had each fraction setup variation less than 3 mm, all three of them with central targets. We applied the measured setup uncertainty and applied their individual variation to generate a new smaller PTV for each case. Their IMRT plans were reoptimized, and as shown in Figure 5, application of patient-specific CTV expansions results shift of DVH curves to the left (arrows), decreasing dose to the brainstem, hippocampus, and temporal lobe. The decrease in mean hippocampus dose would in this case be predicted to preserve neurocognitive function based on dose-volume relationships reported previously [8].

## Discussion

The treatment of intracranial malignancies has been greatly enhanced by the use of radiation [8,9]. The ultimate objective is to administer a dose localized at the target volume coupled with the desired effect of sparing normal tissue, thus, minimizing the likelihood of long-term neurocognitive deficits. Reducing the clinical target volume has been shown to be adequate for tumor control with the intention of diminishing the potential cognitive side effects inherent with radiation therapy [10]. The use of three-dimensional conformal radiation therapy has been effective in increasing tumor control and in reducing acute side effects [11].

The determination of the setup uncertainty prior to the initiation of radiation plays an important role in the treatment of CNS malignancies. Daily setup variation may be underrecognized and may have an adverse impact, including target underdose and normal structures receiving a higher dose than anticipated [12,13]. Daily localization based on cone beam CT imaging may reduce the required setup margin lessening normal tissue exposure to radiation [14]. Patient motion during treatment may affect the dose to critical structures and, therefore, establishing risk volumes are recommended to accurately ascertain the dose administered to normal tissues [3].

Closely monitoring the target volume is vital during radiation therapy to ensure that the doses to the target and normal tissues are not altered which may have deleterious repercussions [15]. Beltran et al. have demonstrated that the target volume during radiation therapy in craniopharyngioma may increase and decrease (-20.7% to 82%), stressing that surveillance imaging is necessary to determine the appropriate dose to the target volume while at the same time closely observing the amount to the normal tissue [15]. Beltran et al. have also recommended daily localization which decreases the PTV margin and minimizes insufficient tumor coverage due to setup uncertainties [2]. In addition, a decrease in the CTV margin lessens the dose directed to normal cerebral tissues [2].

Several studies have addressed daily setup variability in the treatment of head and neck cancer patients using conventional mask immobilization. Hong et al. showed a 3.33 mm absolute average daily setup error in any single dimension while other studies have reported a range of 3–5 mm in head and neck setup variation using weekly portal film measurements [12,16,17]. The patients in the current study were either immobilized with an aquaplast mask and either a base plate or S-frame. In our study, immobilization with an S-frame significantly increased setup uncertainty (3D p < 0.001) primarily in the vertical direction, consistent with flexion of the insert as it protrudes beyond the head of the table. Beltran et al. reported that the setup margin was smaller for those patients who were treated under anesthesia [4]. In the present study, setup uncertainty was statistically significant in patients who received sedatives (3D p < 0.001). We hypothesize that patients at risk for claustrophobia requiring oral sedation in fact would have demonstrated larger variation without sedation, and in our study the sedation was unable to completely ameliorate their anxiety. Patients prescribed either steroids or anti-seizure medications demonstrated no statistical difference in setup uncertainty.

We have shown that there was no statistical difference in setup uncertainty between CBCT and OBI imaging modalities. In addition, setup uncertainty was not significant for the first three days of treatment versus all subsequent treatment days via CBCT guidance. The first three days of CBCT predicted setup uncertainty for subsequent treatments and permitted customized CTV to PTV expansion. Patient weight gain of greater than ten pounds was not associated with increased setup uncertainty. The data demonstrated that patients with a tumor located in the temporal lobe experienced greater setup uncertainty whereas those with pituitary tumors showed the least setup uncertainty.

## Conclusion

Setup uncertainty in the course of radiation treatment may be influenced by several factors, including the immobilization device utilized in therapy, specific medications, and certain tumor locale. To minimize the setup uncertainty, various institutions have instituted a policy of daily and continuous evaluation of interfraction and intrafraction motion for all head and neck cancer patients undergoing intensity-modulated radiation therapy [12,18].

Image-guidance potentially maximizes the therapeutic index of brain irradiation by minimizing setup uncertainty. Custom CTV to PTV expansion results in a reduced dose administered to the OAR which diminishes the possibility of developing neurocognitive, learning, and neuroendocrine deficits.

## Competing interests

The authors declare that they have no competing interests.

## Authors’ contributions

LBES performed the literature search, analyzed the primary data, and played the primary role in the writing of the manuscript. JMC collected the primary data from the treatment machines and developed the spreadsheet. CD and MR were dosimetrists who were involved with the replanning. KS analyzed the primary data. TWV was the neurosurgeon who performed the tumor surgical extraction and wrote the IRB document. ACS was the radiation oncologist who designed the study, collected and analyzed the primary data, administered the radiation to the patients, and wrote the manuscript. All authors read and approved the final manuscript.

## References

[B1] International Commission on Radiation Units and Measurements ICRU Report 50: Prescribing, recording, and reporting photon beam therapy 1993 Bethesda, MD: ICRU

[B2] Beltran C Naik M Merchant TE Dosimetric effect of setup motion and target volume margin reduction in pediatric ependymoma Radiother Oncol 2010 96 2 216 222 10.1016/j.radonc.2010.02.031 20347495

[B3] Beltran C Trussell J Merchant TE Dosimetric impact of intrafractional patient motion in pediatric brain tumor patients Med Dosim 2010 35 1 43 48 10.1016/j.meddos.2009.01.004 19931014 PMC3800030

[B4] Beltran C Krasin MJ Merchant TE Inter- and intrafractional positional uncertainties in pediatric radiotherapy patients with brain and head and neck tumors Int J Radiat Oncol Biol Phys 2011 79 4 1266 1274 10.1016/j.ijrobp.2009.12.057 20605345 PMC3536549

[B5] Cho BC Cho BC van Herk M Mijnheer BJ Bartelink H The effect of set-up uncertainties, contour changes, and tissue inhomogeneities on target dose-volume histograms Med Phys 2002 29 10 2305 2318 10.1118/1.1508800 12408305

[B6] Rahimian J Chen JC Rao AA Girvigian MR Miller MJ Greathouse HE Geometrical accuracy of the Novalis stereotactic radiosurgery system for trigeminal neuralgia J Neurosurg 2004 101 Suppl 3 351 355 15537189

[B7] O'Gorman RL Jarosz JM Samuel M Clough C Selway RP Ashkan K CT/MR image fusion in the postoperative assessment of electrodes implanted for deep brain stimulation Stereotact Funct Neurosurg 2009 87 4 205 210 10.1159/000225973 19556830

[B8] Merchant TE Kun LE Wu S Xiong X Sanford RA Boop FA Phase II trial of conformal radiation therapy for pediatric low-grade glioma J Clin Oncol 2009 27 22 3598 3604 10.1200/JCO.2008.20.9494 19581536 PMC3525947

[B9] Dunbar SF Tarbell NJ Kooy HM Alexander E III Black PM Barnes PD Goumnerova L Scott RM Pomeroy SL La VB Stereotactic radiotherapy for pediatric and adult brain tumors: preliminary report Int J Radiat Oncol Biol Phys 1994 30 3 531 539 10.1016/0360-3016(92)90938-E 7928483

[B10] Merchant TE Kiehna EN Kun LE Mulhern RK Li C Xiong X Boop FA Sanford RA Phase II trial of conformal radiation therapy for pediatric patients with craniopharyngioma and correlation of surgical factors and radiation dosimetry with change in cognitive function J Neurosurg 2006 104 2 Suppl 94 102 16506496 10.3171/ped.2006.104.2.5

[B11] Merchant TE Three-dimensional conformal radiation therapy for ependymoma Childs Nerv Syst 2009 25 10 1261 1268 10.1007/s00381-009-0892-9 19373477

[B12] Hong TS Tome WA Chappell RJ Chinnaiyan P Mehta MP Harari PM The impact of daily setup variations on head-and-neck intensity-modulated radiation therapy Int J Radiat Oncol Biol Phys 2005 61 3 779 788 10.1016/j.ijrobp.2004.07.696 15708257

[B13] Kumar S Burke K Nalder C Jarrett P Mubata C A'hern R Humphreys M Bidmead M Brada M Treatment accuracy of fractionated stereotactic radiotherapy Radiother Oncol 2005 74 1 53 59 10.1016/j.radonc.2004.06.008 15683670

[B14] Beltran C Pai Panandiker AS Krasin MJ Merchant TE Daily image-guided localization for neuroblastoma J Appl Clin Med Phys 2010 11 4 3388 21081896 10.1120/jacmp.v11i4.3388 PMC5720396

[B15] Beltran C Naik M Merchant TE Dosimetric effect of target expansion and setup uncertainty during radiation therapy in pediatric craniopharyngioma Radiother Oncol 2010 97 3 399 403 10.1016/j.radonc.2010.10.017 21074883

[B16] Menke M Hirschfeld F Mack T Pastyr O Sturm V Schlegel W Photogrammetric accuracy measurements of head holder systems used for fractionated radiotherapy Int J Radiat Oncol Biol Phys 1994 29 5 1147 1155 10.1016/0360-3016(94)90412-X 8083085

[B17] Rabinowitz I Broomberg J Goitein M McCarthy K Leong J Accuracy of radiation field alignment in clinical practice Int J Radiat Oncol Biol Phys 1985 11 10 1857 1867 10.1016/0360-3016(85)90046-X 4044349

[B18] Tome WA Meeks SL McNutt TR Buatti JM Bova FJ Friedman WA Mehta M Optically guided intensity modulated radiotherapy Radiother Oncol 2001 61 1 33 44 10.1016/S0167-8140(01)00414-5 11578726

